# Supplementary material for: PI3Kδ hyper-activation promotes development of B cells that exacerbate Streptococcus pneumoniae infection in an antibody-independent manner
Source: Nat Commun. 2018 Aug 9;9:3174. doi: 10.1038/s41467-018-05674-8 (PMC6085315; doi:10.1038/s41467-018-05674-8)
Supplement: Supplementary file 1 — Supplementary Information [file 41467_2018_5674_MOESM1_ESM.pdf]

Stark A et al:

PI3Kδ hyper-activation promotes development of B cells that exacerbate Streptococcus pneumoniae infection in an antibody-independent manner.

Supplementary Figures 1-10.

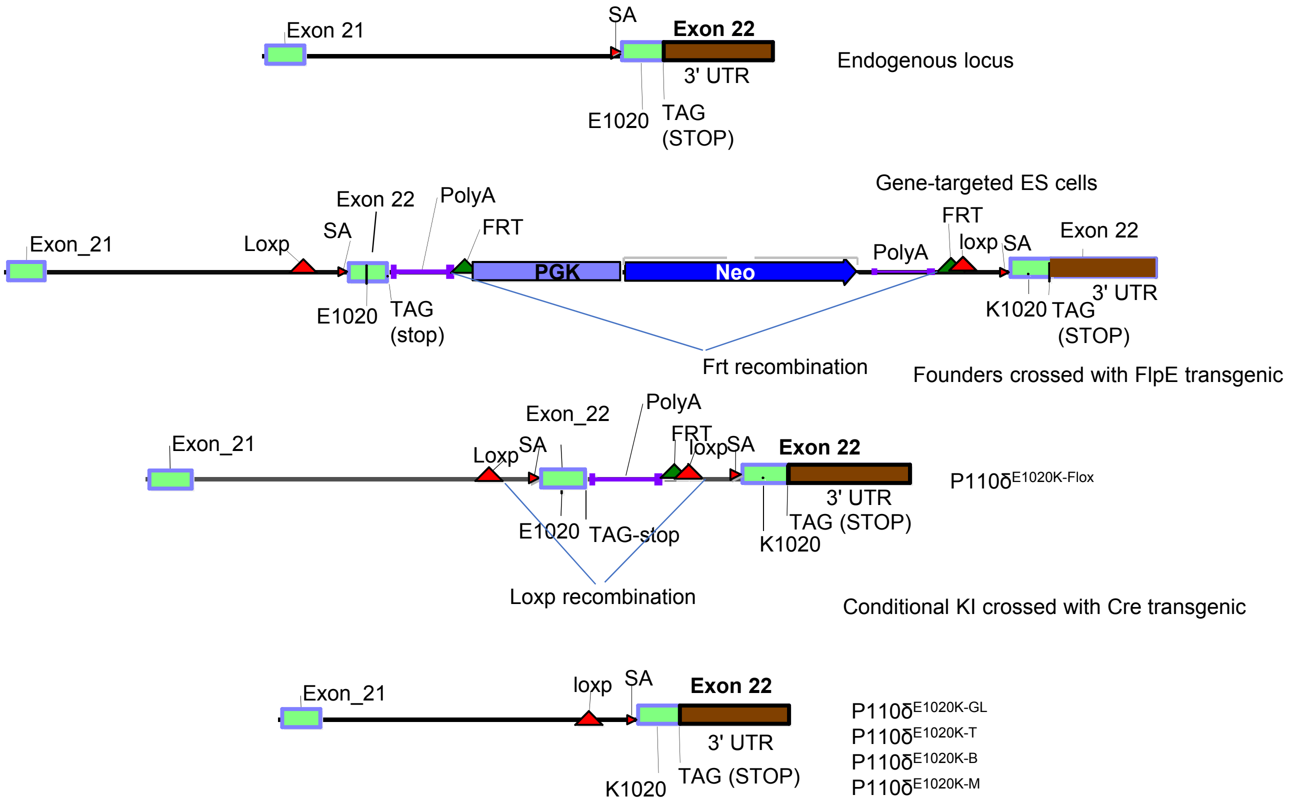

Supplementary Figure 1: Gene targeting strategy for generating conditional p110δ^{E1020K} mice
The p110δ^{E1020K} mice were generated by OzGene using homologous recombination in ES cells. A duplicate sequence corresponding to the last coding exon in *Pik3cd* was flanked by loxP sites and inserted 3' to the original sequence. The original sequence encoding E1020 was mutated to K1020. Upon Cre-mediated recombination, the wild-type sequence is replaced by the mutant E1020K sequence. In this study, we used *Tnfrsf4*^{cre} to delete in the germline, *Cd4*^{cre} to delete in T cells, *Mb1*^{cre} to delete in B cells and *Lyz2*^{Cre} to delete in myeloid cells.

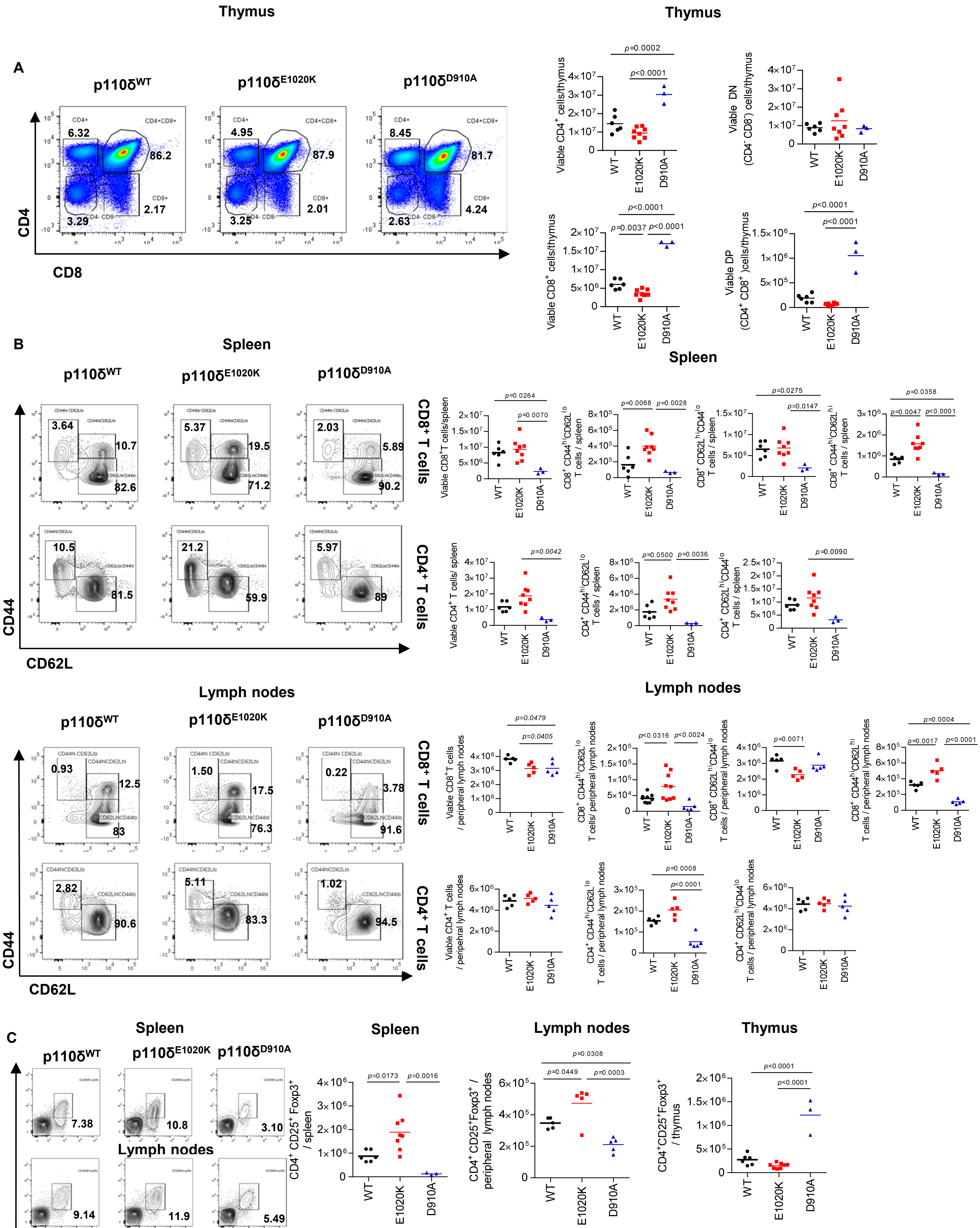

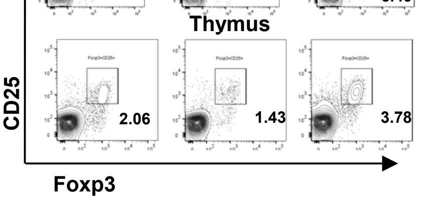

Supplementary figure 2: Phenotype of T cells in p110δ$^{E1020K-GL}$ mice.
T cell populations in the thymus, spleen and lymph nodes of wild-type, p110δ$^{E1020K-GL}$ and p110δ^{D910A} mice were analyzed by flow cytometry. Representative pseudocolor or contour plots with outliers show mean cell proportions. **A:** Thymic T cell numbers in p110δ^{E1020K} mice were normal apart from a reduction in single positive CD8$^+$ cells. **B:** The number of activated CD4$^+$ and CD8$^+$ T cells (CD44^{hi}CD62^{lo}) as well as memory CD8$^+$ T cells (CD44^{hi}CD62L^{hi}) in lymph nodes and spleen were increased in p110δ^{E1020K} mice. **C:** The number of CD4$^+$CD25$^+$Foxp3$^+$ regulatory T cells was increased in the lymph nodes and spleen of p110δ^{E1020K} mice. (Mean cell numbers are shown; representative data from 2 independent experiments; Thymus and spleen: wild-type n=7, p110δ$^{E1020K-GL}$ n=8; p110δ^{D910A} n=3, Lymph nodes: wild-type n=5, p110δ$^{E1020K-GL}$ n=5; p110δ^{D910A} n=5; data-points represent individual animals)

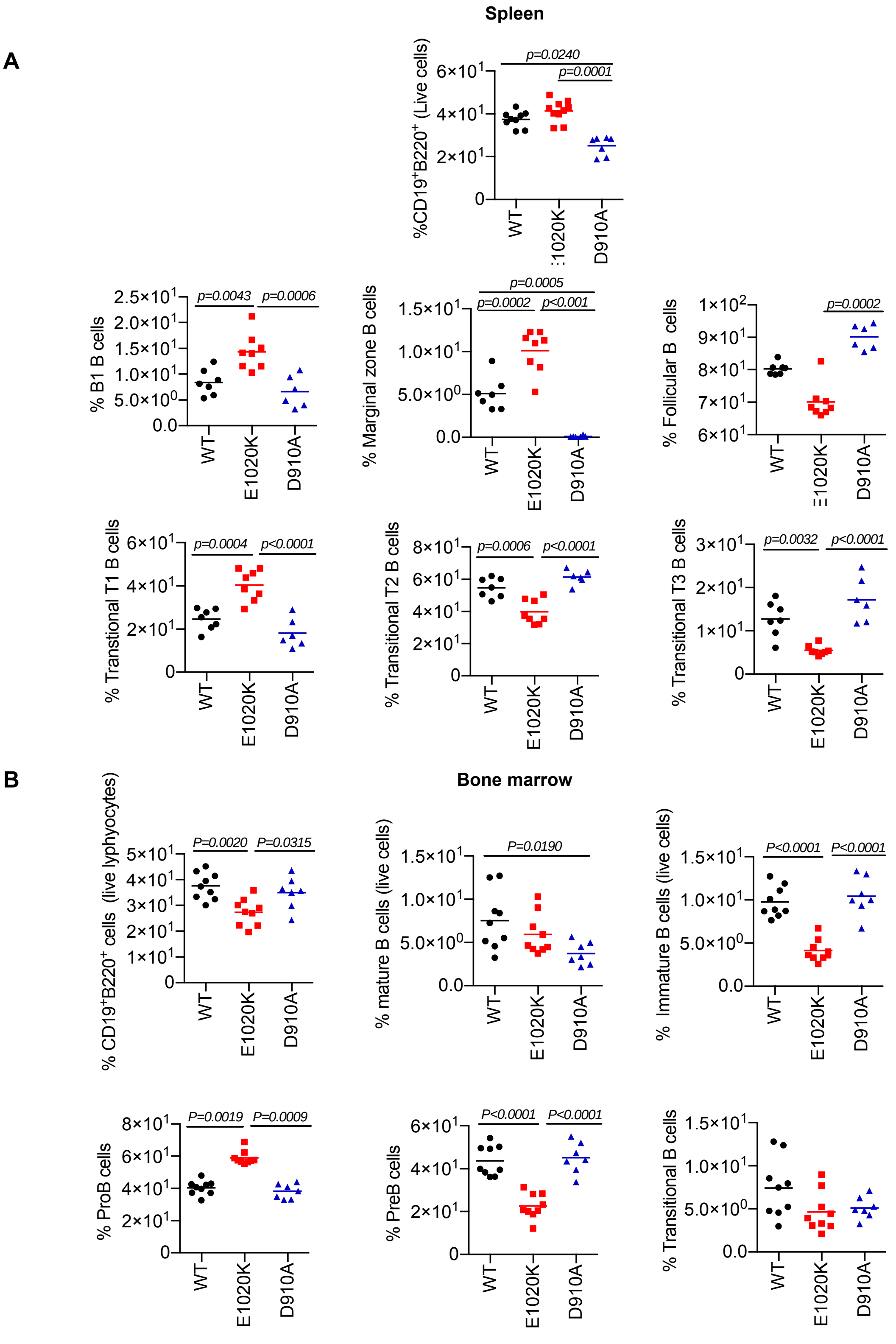

Supplementary figure 3: Phenotype of B cells in p110δ$^{E1020K-B}$ mice.

B cell subsets in the spleen and bone marrow from wild-type, p110δ$^{E1020K-B}$ and p110δ^{D910A} mice were analyzed by flow cytometry, and was shown to recapitulate the phenotype of p110δ$^{E1020K-GL}$ mice. **A:** In the spleen, the proportion of B1, marginal zone (MZ) and T1 transitional cells were increased, while the proportion of follicular B cells were reduced. **B:** Analysis of the bone marrow showed increased proportions of pro-B cells in p110δ$^{E1020K-B}$ mice with reduced proportions of pre-B cells and immature B cells, and a trend towards reduced proportions of transitional and mature B cells. Populations of cells are described as follows: Splenic B cells - Total B cells $CD19^{+}B220^{+}$, B1 cells $CD19^{+}B220^{+}CD23^{-}$CD21-, Follicular B cells $CD19^{+}B220^{+}CD23^{+}CD21^{+}$, Marginal zone B cells $CD19^{+}B220^{+}CD23^{-}CD21^{+}$, Transitional T1 B cells $B220^{+}CD93^{+}IgM^{+}CD23^{-}$, Transitional T2 B cells $B220^{+}CD93^{+}IgM^{+}CD23^{+}$, Transitional T3 B cells $B220^{+}CD93^{+}IgM^{-}CD23^{-}$; Bone marrow B cells - Immature B cells $CD19^{+}B220^{lo}IgM^{+}$, Mature B cells $CD19^{+}B220^{hi}IgM^{+}$, Pro-B cells $B220^{+}IgM^{-}CD19^{+}CD25^{-}$, Pre-B cells $B220^{+}$IgMCD19^{+}CD25^{+}, Transitional B cells $B220^{+}IgD^{+}$. (Mean cell proportions are shown; combined data from 2 independent experiments; Spleen: wild-type n=7, p110δ$^{E1020K-B}$ n=8; p110δ^{D910A} n=6; Bone Marrow: wild-type n=9, p110δ$^{E1020K-B}$ n=9; p110δ^{D910A} n=7. Data-points represent individual animals).

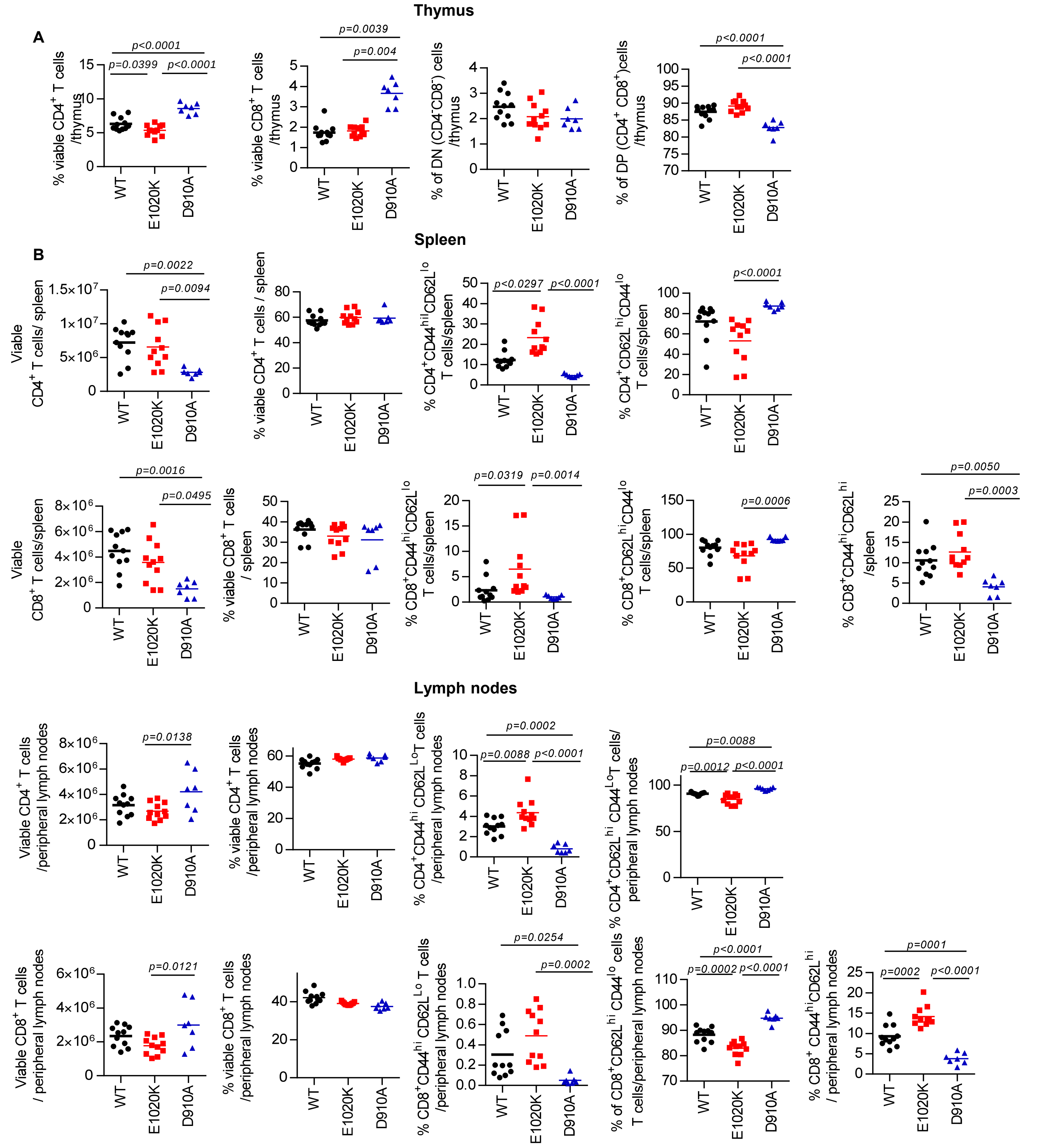

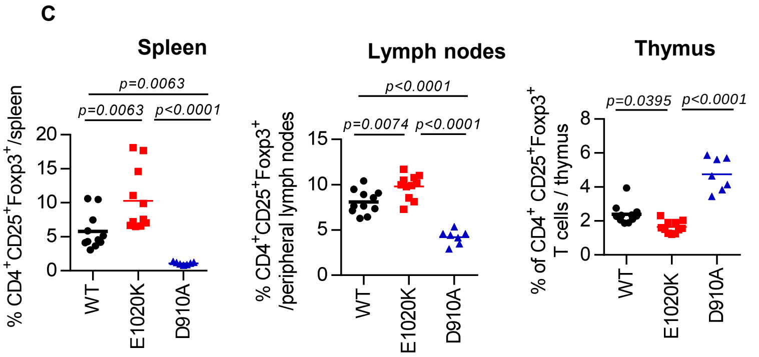

Supplementary figure 4: Phenotype of T cells in p110δ$^{E1020K-T}$ mice.

T cell populations in the thymus, spleen and lymph nodes in wild-type, p110δ$^{E1020K-T}$ and p110δ^{D910A} were analyzed by flow cytometry. **A:** Thymic T cell proportions in p110δ$^{E1020K-T}$ mice were normal **B:** In the spleen, the proportions of activated CD4$^+$ and CD8$^+$ T cells (CD44^{hi}CD62^{lo}) were increased, and there was a trend towards increased proportions of memory CD8$^+$ T cells (CD44^{hi}CD62L^{hi}) in p110δ$^{E1020K-T}$ mice. In the lymph nodes of p110δ$^{E1020K-T}$ mice, the proportions of activated CD4$^+$ T cells (CD44^{hi}CD62^{lo}) were increased with a trend towards increased proportions of activated CD8$^+$ T cells, as well as increased proportions of memory CD8$^+$ T cells (CD44^{hi}CD62L^{hi}). **C:** The proportion of CD4$^+$CD25$^+$Foxp3$^+$ regulatory T cells were increased in the lymph nodes and spleen of p110δ$^{E1020K-T}$ mice. (Mean cell proportion/number is shown; combined data from 3 independent experiments; wild-type n=11, p110δ$^{E1020K-T}$ n=11, p110δ^{D910A} n=7; data-points represent individual animals).

.

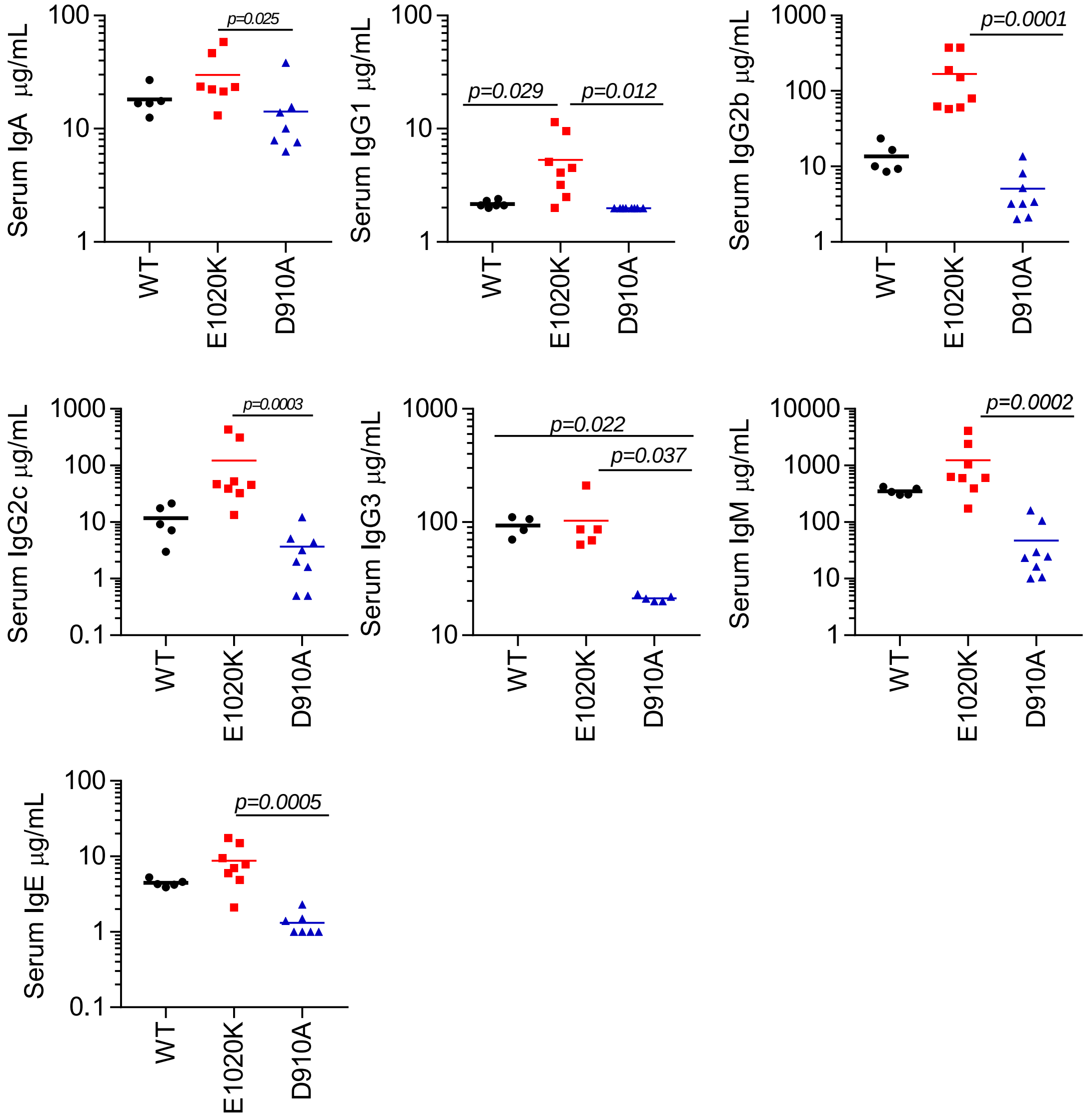

Supplementary figure 5: p110δ^{D910A} mice are deficient in IgA, IgG, IgM and IgE serum immunoglobulins
Analysis of serum immunoglobulins from naïve mice (age 8-12 weeks) showed significantly increased levels of IgG1 and IgG2b, and a trend towards increased levels IgG2c, IgM, IgA, and IgE in p110δ$^{E1020K\text{-}GL}$ mice, while IgG3 levels were similar compared to wild-type mice. p110δ^{D910A} mice were antibody deficient for all isotypes analyzed. (Mean antibody levels are shown; wild-type n=5, p110δ$^{E1020K\text{-}GL}$ n=7, p110δ^{D910A} n=7; data-points represent individual animals).

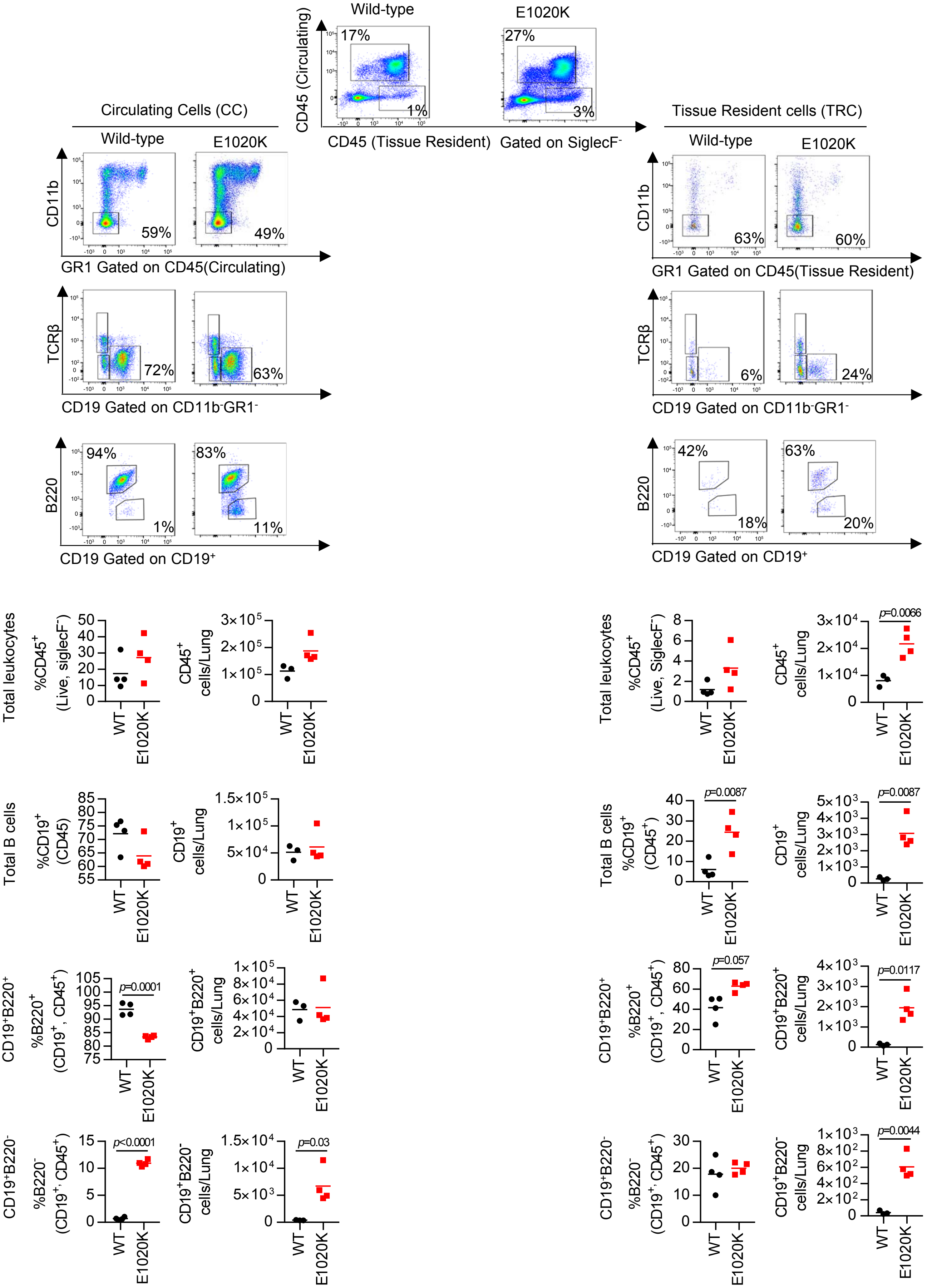

Supplementary Figure 6: Tissue resident B cell numbers are increased in the lungs of naïve p110δ$^{E1020K\text{-}GL}$ mice
Biotin-conjugated anti-CD45 was injected intravenously to distinguish between circulating (CC) and tissue resident (TRC) cells in the lungs of naïve mice. p110δ$^{E1020K\text{-}GL}$ mice showed an increase in the proportion and number of tissue resident leukocytes (CD45$^+$TRC), but not circulating leukocytes. There was also an increase in the proportion and number of total CD19$^+$ B cells among the tissue resident cell population. Among circulating leukocytes (CD45$^+$IV), total CD19$^+$ B cell numbers were similar in p110δ^{WT} and p110δ^{E1020K} mice, and p110δ^{E1020K} mice had increased numbers and proportions of CD19$^+$B220$^-$ B cells. (Mean values shown; representative data from 2 independent experiments n=4; data-points represent individual animals).

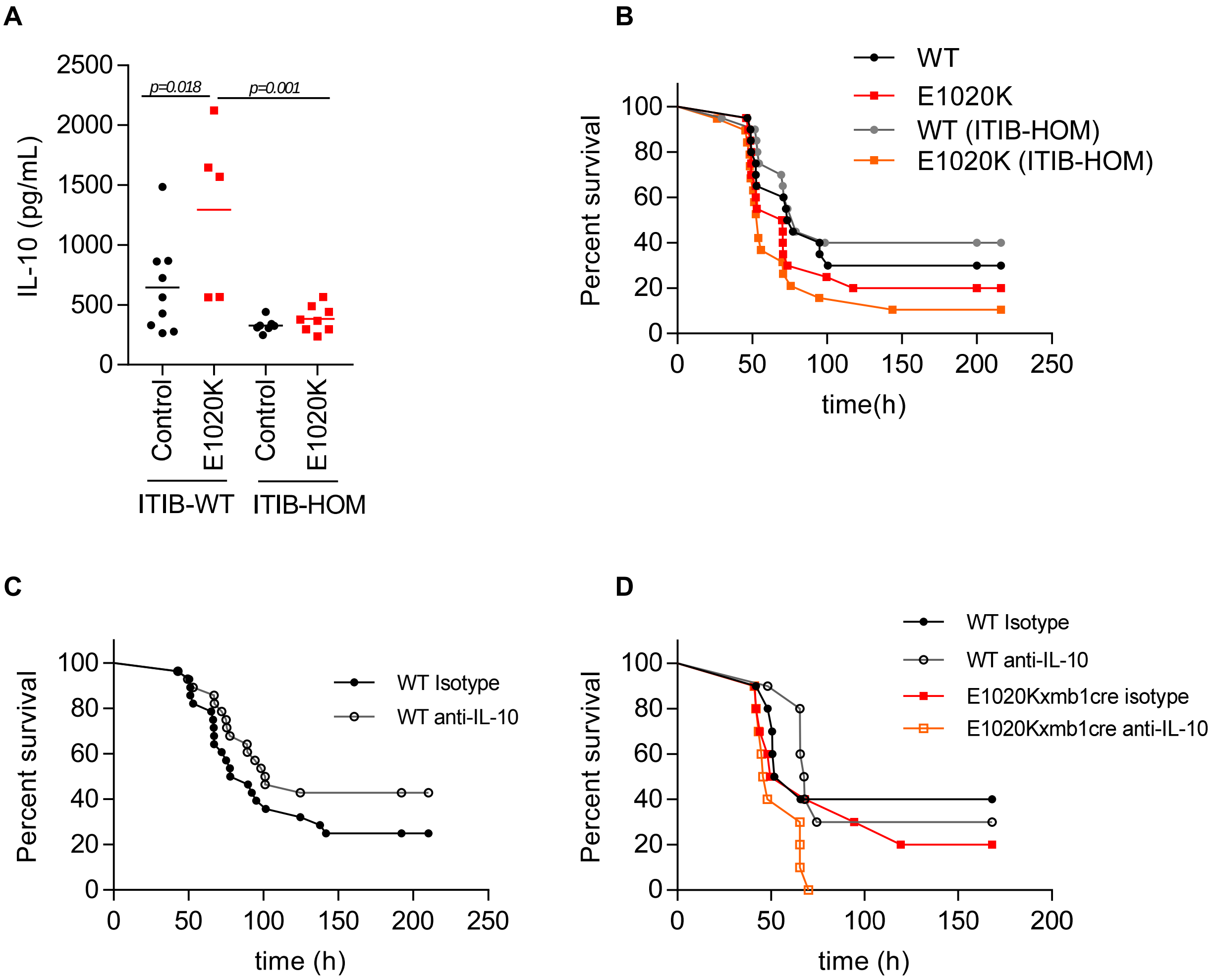

Supplementary Figure 7: Systemic IL-10 downregulation or local depletion does not improve the outcome of *S. pneumoniae* infection in p110δ^{E1020K} mice.

Il10^{ITIB} homozygous p110δ^{E1020K} and WT mice as well as *Il10*^{ITIB} wildtype p110δ^{E1020K} and WT mice were infected with *S. pneumoniae* serotype 4, TIGR4 and monitored for disease development. Animals were culled and serum samples collected when the predetermined humane endpoint was reached or at 10 days post-infection for surviving mice. To deplete IL-10 levels locally in the lung, 200µg anti-IL-10 (JES5-2A5) or Isotype control was administered intranasally starting 24h prior to infection with *S. pneumoniae* and continued for the duration of the experiment. **A:** *Il10*^{ITIB} wildtype mice showed higher serum IL-10 levels compared to *Il10*^{ITIB} homozygous mice, confirming that these animals are IL-10 hypomorphic. **B:** *Il10*^{ITIB} homozygous mice did not have an improved outcome to *S. pneumoniae* infection compared to *Il10*^{ITIB} wildtype mice, despite lower systemic levels of IL-10. **C:** Wildtype mice showed a non-significant trend towards improved outcome with anti-IL-10 treatment. **D:** anti-IL-10 treatment did not improve disease outcome following *S. pneumoniae* infection in B cell conditional p110δ$^{E1020K-B}$ mice. (Data-points represent individual animals. A: mean values are shown, n=5-9; B-C: Combined data from two independent studies, n=20 (B), n=24(C); D: Data from one study, n=10)

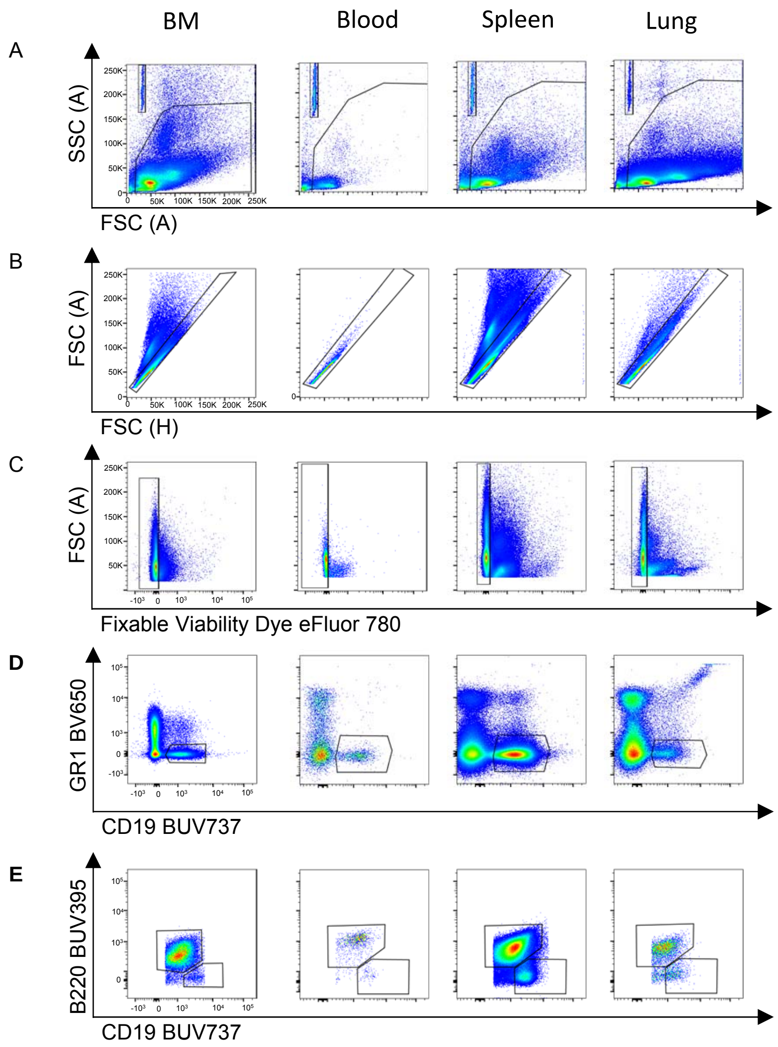

Supplementary figure 8: Gating strategy for the identification of B220⁻ B cells

B220⁻ B cells were identified in the bone marrow, blood, spleens and lungs from wildtype and p110δ^{E1020K} mice by flow cytometry. Representative pseudocolor plots from p110δ^{E1020K} mice show the gating strategy used to identify B220⁻ B cells. **A:** Counting beads (high side scatter, low forward scatter) and cells were identified by side scatter and forward scatter properties**. B:** From the cell gate, single cells were identified based on forward scatter peak height and width parameters. **C:** From the single cell gate, live cells were identified by low binding of an amine reactive fixable viability dye. **D:** From the live cell gate, CD19⁺ total B cells were identified. **E:** From the total CD19⁺ B cell gate, B220⁺ and B220⁻ CD19⁺ B cells were identified.

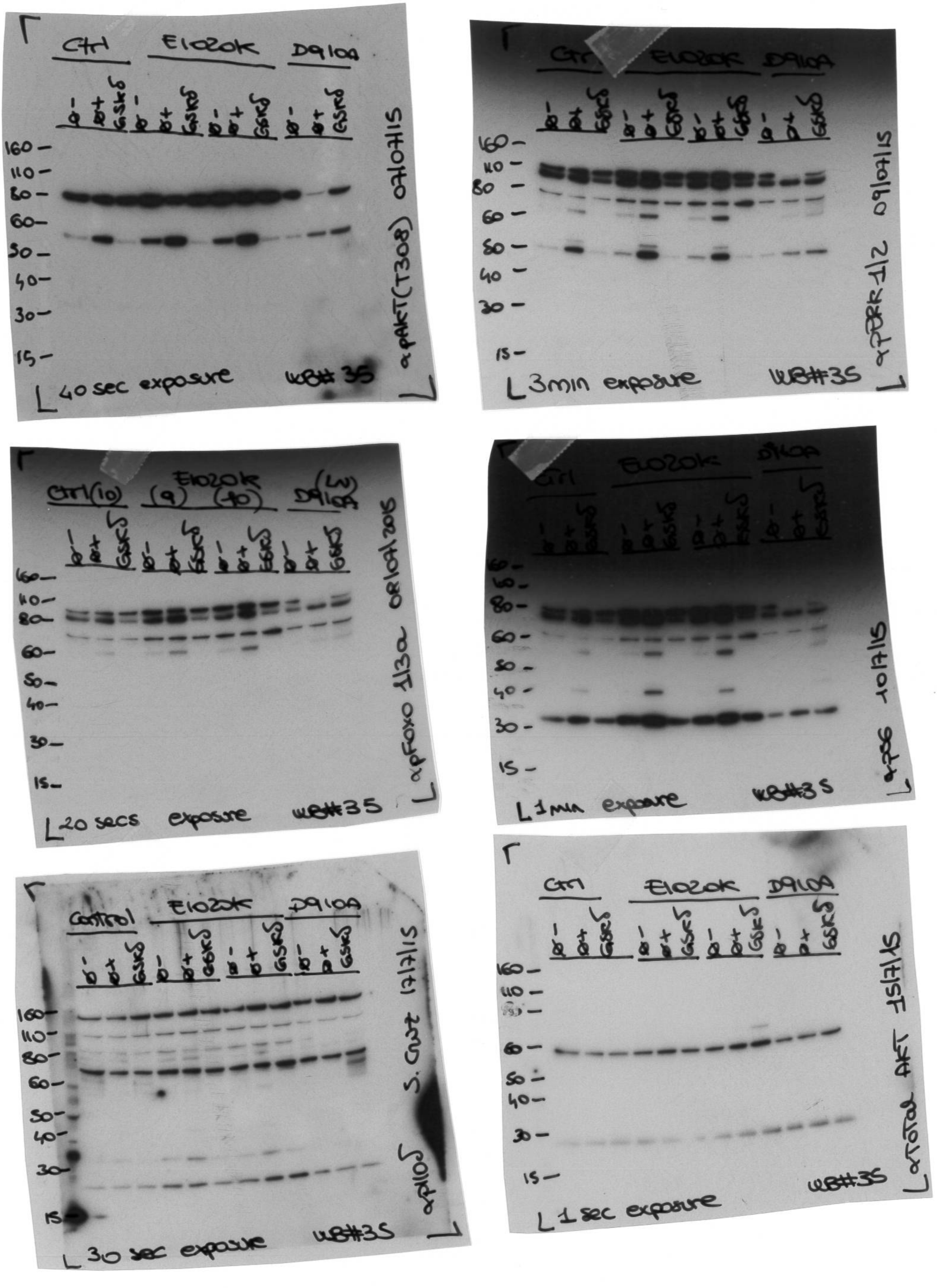

Supplementary figure 9 (blots used for Fig 2c).

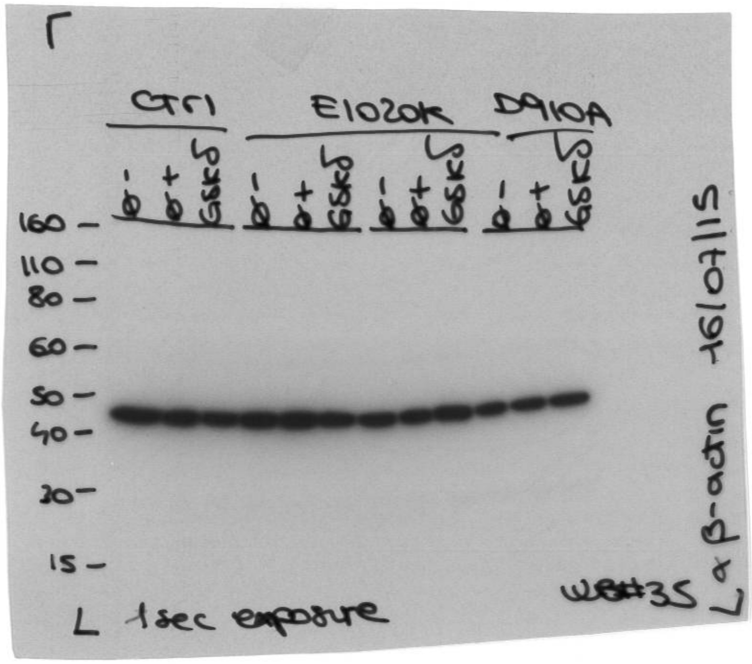

Supplementary figure 9 (continued, blots used for Fig 2c).

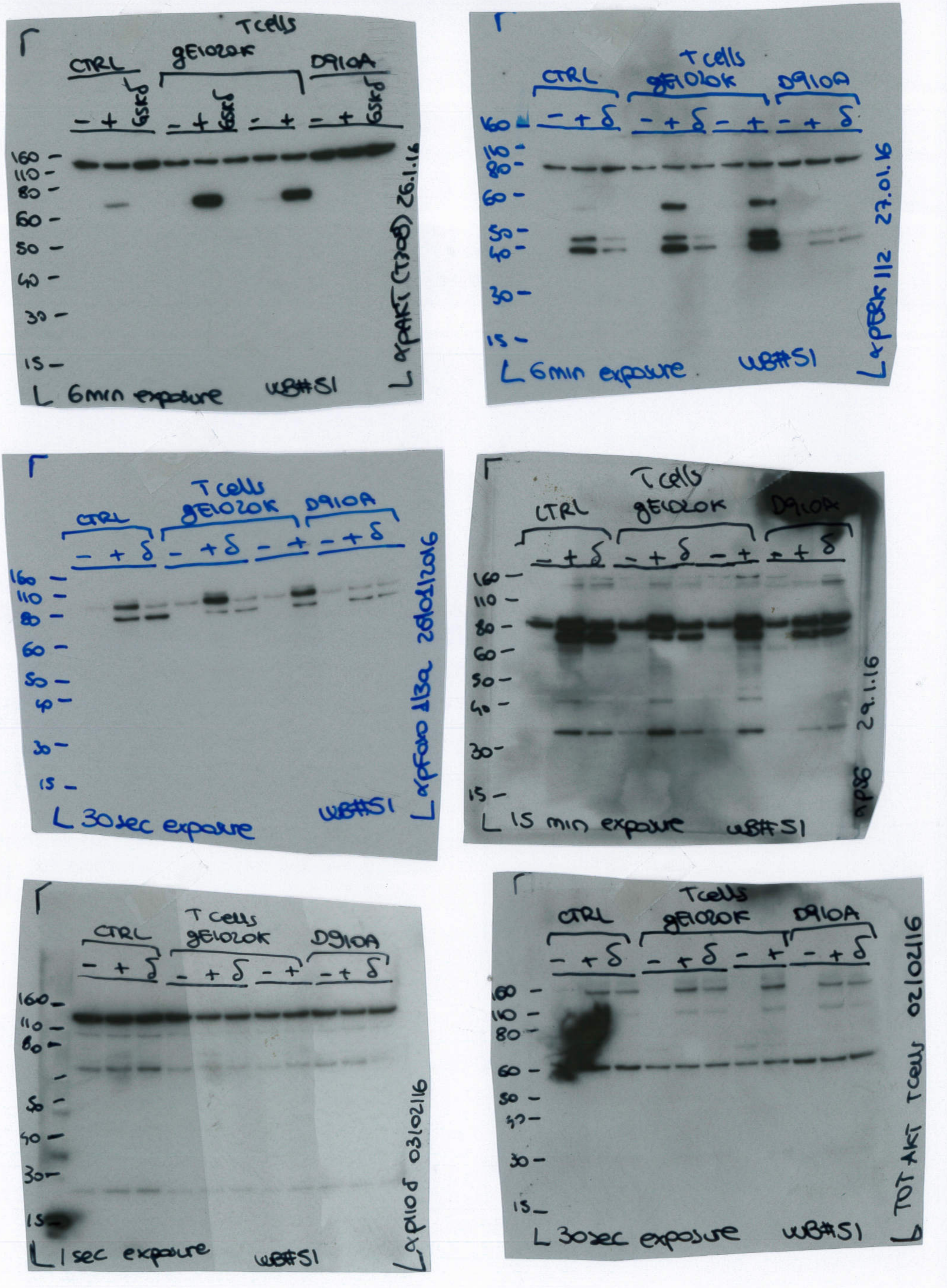

Supplementary figure 10 (blots used for Fig 2c).

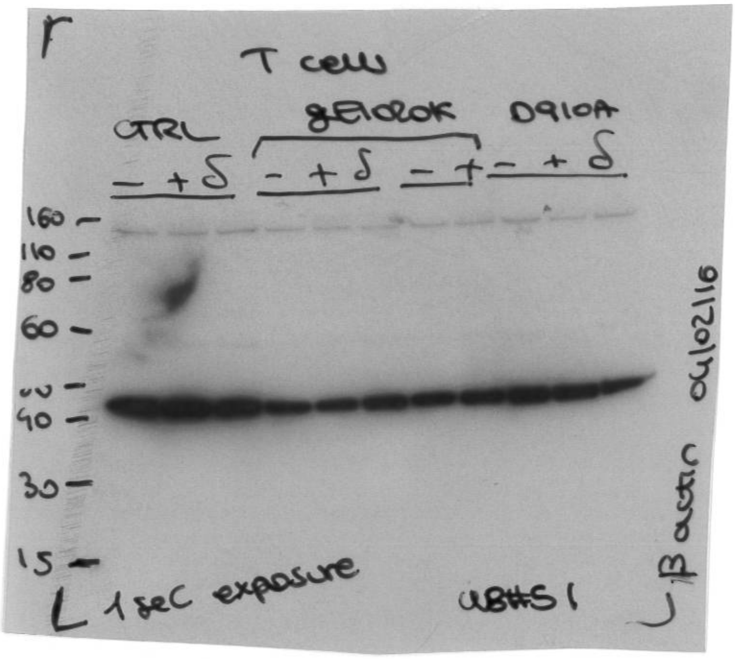

Supplementary figure 10 (continued, blots used for Fig 2d).
